# Comparing the Role of Aberrant Salience and Dissociation in the Relation between Cumulative Traumatic Life Events and Psychotic-Like Experiences in a Multi-Ethnic Sample

**DOI:** 10.3390/jcm8081223

**Published:** 2019-08-15

**Authors:** Deidre M. Anglin, Adriana Espinosa, Bassem Barada, Rona Tarazi, Ashley Feng, Rachel Tayler, Neil M. Allicock, Supriya Pandit

**Affiliations:** 1 Department of Psychology, The City College, City University of New York (CUNY), New York, NY 10031, USA; 2 Doctoral Psychology Programs, The Graduate Center of CUNY, New York, NY 10031, USA

**Keywords:** trauma, psychotic-like experiences, ethnic, aberrant salience, dissociation

## Abstract

Exposure to traumatic life events (TLE) is a risk factor for psychosis. Yet, a dearth of studies examines factors linking TLE to psychosis, while considering differences in TLE frequency. This study investigated dissociation and aberrant salience as mediators and moderators of the relation between three TLE groups (none, 1–3 TLE and 4+ TLE) and psychotic-like experiences (PLE) in a multi-ethnic sample of 816 emerging adults. The participants completed self-report inventories of PLE (Prodromal Questionnaire), TLE (Life Events Checklist), dissociative experiences (Dissociative Symptoms Scale), and aberrant salience (Aberrant Salience Inventory). As expected, groups with higher TLE frequency endorsed higher PLE. Parallel mediation models indicated that, while aberrant salience mediated the relation between both levels of cumulative traumatic experiences (1–3 TLE and 4+ TLE) and PLE, dissociation only mediated the relation between exposure to at least four different traumatic experiences and PLE. The moderation results showed that risk of PLE was highest among individuals with 1–3 TLE who endorsed dissociation. Our results suggest that, while aberrant salience processing explains why TLE may relate to higher psychosis risk, dissociation’s role in this relation is dependent on the number of different TLE exposures.

## 1. Introduction 

### 1.1. Traumatic Life Events and Psychosis

Studies consistently find that exposure to traumatic life events (TLE) is an important environmental risk factor in the development of psychosis [1,2]. For an extensive review on this relationship, see Gibson et al. [3]. This relationship has also been demonstrated across the entire psychotic spectrum, including for psychotic-like experiences (PLE) [4,5,6]. A meta-analysis of TLE occurring during adulthood found that TLE increased the risk for psychotic experiences (2–7 times) in non-treatment-seeking general population samples [7]. A study conducted by Croft, Heron, Teufel, et al. [2] showed that, up to the age of 17 years, exposure to any trauma across several developmental periods studied was associated with increased odds of psychotic experiences at age 18. The results also suggested an increase in effect size with exposure to a greater number of trauma types (e.g., three or more types of trauma exposure was associated with a 4.7-fold increase in odds of psychotic experiences) [2]. 

### 1.2. Dissociation as a Mediating Factor

While the relationship between trauma and psychosis is well established, the mechanism by which it operates, especially in diverse populations, remains unclear. Empirical evidence highlights dissociation as a salient mediating factor [8]. Dissociation is broadly defined by three domains: (1) positive dissociation: a loss of continuity in subjective experience, accompanied with unwanted intrusions into awareness and behavior; (2) negative dissociation: an experienced loss of control over mental functions, such as gaps in memory and self-identification; and, (3) a perceived sense of disconnectedness, which may include distorted perceptions of the self and the social environment [9]. Research demonstrates that dissociation is moderately associated with trauma exposure and severity, in that more dissociative symptoms occur with more frequent trauma-related psychiatric diagnoses [10]. While disconnecting from the environment attempts to preserve an individual’s core sense of safety and stability, more disruptive dissociative symptoms will likely emerge over time [11]. 

Empirical research indicates that dissociation mediates the relation between early childhood maltreatment, particularly childhood sexual abuse, and hallucination-proneness in a sample of Spanish patients on the psychosis spectrum [12,13], and delusional ideation in a sample of non-clinical United Kingdom (U.K.) undergraduate students [14]. Dissociation was similarly highlighted as a potential mediator between childhood trauma and hallucinations, though not between childhood trauma and delusions [15]. Other inconsistencies include research that suggests dissociation does not mediate the relation between trauma and PLE consistently across multiple ethnic groups [16], with dissociation being particularly explanatory for the Black subgroup of young adults, with little to no relevance for the Asian American subgroup [16]. 

### 1.3. Aberrant Salience as a Mediating Factor 

Aberrant salience is another important factor that has received less empirical attention as a potential mediating factor. The aberrant salience model of psychotic symptoms proposes that hallucinations and delusions may arise from the inappropriate assignment of salience to thoughts and perceptions that would typically be considered less significant or personally relevant [17,18,19,20,21]. The degree to which an individual assigns importance to stimuli signifies the motivational properties that attract the individual’s attention to it [22]; the degree to which the stimuli is rendered as appetitive or aversive [23]; and, the stimuli’s salience as a motivating factor for inappropriately influencing behavior [24]. Along with these abnormal perceptual experiences, these individuals usually report feeling that their senses have become enhanced, and their attention to details sharpened. Aberrant salience is thought to occur when individuals have more elevated than normal presynaptic dopaminergic activity [25,26], which is supported by similar findings of heightened dopamine levels in schizophrenia and individuals that are vulnerable to developing psychosis [27,28]. Dysregulation in these dopamine-rich salience brain networks results in everyday misfires of salience attribution, i.e., psychotic symptoms. Very few studies have examined whether or not dysruption in this salience network could explain why individuals that are exposed to trauma might be more vulnerable to psychotic phenomenonlogy, but Howes and Murray [29] suggest that traumatic event expsoures may trigger these aberrant moments of information processing and Gaweda et al. [30] recently found that aberrant salience mediated the relation between childhood cumulative trauma and neglect and PLE. It is not clear whether this aberrant information processing would also explain why exposure to traumatic life events, more generally, is related to increased PLE. 

The purpose of this study is to compare the relative importance of dissociation and aberrant salience processes in the relations between number of different traumatic life event exposures and PLE. Particularly, the first aim is to assess the extent to which levels of cumulative traumatic experiences (none, 1–3 TLE, 4+ TLE) are indirectly associated with PLE through dissociation and/or aberrant salience. The second aim is to determine whether the relations between different levels of cumulative traumatic experiences and PLE are moderated by dissociation and/or aberrant salience. 

## 2. Methods

### 2.1. Participants 

The participants were obtained from a large urban public university system in the northeastern United States, whose students reflect the demographics of the surrounding urban communities (i.e., commuter students and a high proportion of racial and ethnic minorities). Undergraduate students from various courses in multiple disciplines were recruited through an online participant recruitment website and were deemed to be eligible if they were emerging adults (i.e., age 18–29) and self-identified as Black/African American/African descent or as a first- or second-generation immigrant. This was meant to maximize the recruitment of young racial and ethnic minority adults possessing characteristics that have been implicated in psychosis risk [31]. A total of 816 participants completed a battery of self-report questionnaires, including, but not limited to, those that are described below, on computers in a research lab. The University Institutional Review Board approved the study protocol. Written informed consent was obtained from all of the participants and course credit was given for their participation.

### 2.2. Measures

**Figure 1:**
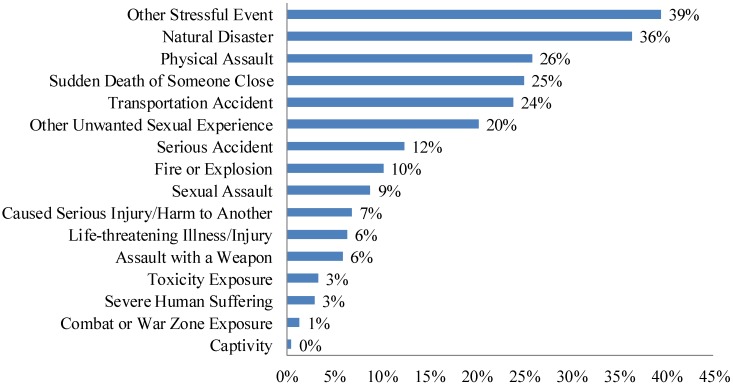
Endorsement of Traumatic Life Events.

Cumulative exposure to traumatic life events (TLE) was evaluated with the 17-item Life Events Checklist (LEC) [32], which is a widely used measure that is used to screen lifetime exposure to events that may induce posttraumatic stress disorder [32]. Specifically, this self-report questionnaire lists 16 experiences related to PTSD (e.g., physical assault, sexual assault, natural disaster, combat, or exposure to a warzone), and one additional item capturing exposure to other significantly stressful events that are not listed among the other items. Participants select whether each event happened directly to them, they witnessed it, they learned about it, they did not know, or it did not apply to them. The scale has demonstrated good test-retest reliability and content validity [32]. For the present study, the LEC variable was calculated by counting the number of responses indicating direct experience of an event. All 17 items on the scale and percentage of respondents endorsing direct experience of each item are indicated in Figure 1. A three-level trauma variable was created from the LEC total count variable, consisting of 0 TLE, 1–3 TLE, and 4 or more TLE experienced directly. This categorization is based on previous research regarding TLE in non-treatment seeking populations [33], which suggests that the 4+ threshold is meaningful for psychosis risk [33,34]. 

The frequency of psychotic-like experiences (PLE) was assessed with the 45-item positive subscale of the Prodromal Questionnaire-Likert (PQ-Likert) [35]. This subscale assesses attenuated (i.e., less frequent, severe, distressing, or convincing) positive psychotic symptoms (i.e., PLE) in the past month while not under the influence of drugs, alcohol, or other medications [35]. The PQ has been found to demonstrate moderate concurrent validity, strong sensitivity, and moderate specificity with other semi-structured interviews that assess for psychosis [21,36]. Positive symptoms have the strongest predictive value for distinguishing a clinically high risk or psychotic clinical syndrome from no syndrome [37]. For the purpose of the present study, each positive symptom that was endorsed by the participant was aggregated into a composite variable to capture a continuum of PLE, which had Cronbach α = 0.92. 

Dissociative symptoms were measured with an earlier unpublished version of the Dissociative Symptoms Scale (DSS) [38]. This 20-item self-report measure is designed to assess the dissociative styles elicited in response to traumatic experiences. The scale was developed to measure the incidence of moderately severe trauma-related intrusions, gaps in awareness or memory, and distortions in perceptions of oneself or one’s surroundings that persist after trauma exposure. The participants were instructed to report how frequently they had experienced each item in the past week on a Likert scale ranging from 0 (not at all) to 4 (more than once a day). The scale consists of four subscales: (a) depersonalization/derealization (b) gaps in awareness and memory, (c) sensory misperception, and (d) cognitive behavioral re-experiencing. The frequency of each item across all subscales was totaled to reflect a grand total dissociation score. The measure has demonstrated excellent internal consistency (Cronbach’s α = 0.91) in a nonclinical university sample and good content validity [38]. The internal consistency for the present study was excellent (Cronbach’s α = 0.93).

Aberrant Salience was measured with the Aberrant Salience Inventory (ASI) [17]. The ASI is a 29-item yes–no questionnaire with five subscales that measure different aspects of the experience of aberrant salience, including feelings of increased significance, sharpening of senses, impending understanding, heightened emotionality, and heightened cognition. Previous research has found that the ASI is highly correlated with other measures of schizotypal personality traits, is elevated in participants at risk for the development of psychotic disorders, and is elevated in inpatients with a history of psychosis as compared with inpatients without a history of psychosis [17]. Moreover, the ASI has discriminant validity from other measures of schizotypal personality traits, as the ASI has been found to be correlated with measures reflecting increased subcortical dopamine, whereas other psychosis proneness measures were not correlated with such measures [17]. At the same time, there is very little, if any, overlap in item content between the types of experiences that are asked about on the ASI and the types of experiences on Perceptual Aberration/Magical Ideation (PerMag) scales or on other PLE scales [17]. The internal consistency for the present study was excellent (Cronbach’s α = 0.90). 

The sociodemographic variables included a four-level race and ethnicity variable: (a) Black (includes individuals born in the United States, Africa, or the Caribbean), (b) Hispanic/Latino, (c) Asian and Asian Pacific Islander, and (d) Other (includes White, Middle Eastern, and Biracial). The other sociodemographic covariates were gender, age (years), and annual family income, which were reported in nineteen categories from zero to $100,000 and over. 

### 2.3. Data Analysis

We verified that the skewness and kurtosis coefficients of study variables were within the bounds that were suggested in the literature [39]. Bivariate tests were used to examine gender, racial and ethnic, age, and income associations with TLE and PLE scores. Independent samples *t* tests or one-way analyses of variance (ANOVA) with Bonferroni corrections in post hoc comparisons were used to assess the mean differences between the groups. Chi-square tests of independence were used to determine the relations between categorical variables. Pearson correlations assessed associations between continuous variables.

Parallel mediation tested whether ASI and dissociation simultaneously mediated the relation between TLE and PLE, while comparing the relative contribution of each mediator. The indirect effects were obtained while using 5000 bootstrapped replications, which yielded 95% bias corrected confidence intervals (CI). Mediation is confirmed if the confidence intervals do not contain zero. These models controlled for age, gender, income, and racial and ethnic group. The moderating roles of dissociation and ASI on the relation between TLE and PLE were assessed using two separate multicategorical hierarchical regressions of two levels each. In each regression, the moderator, age, gender, income, and racial and ethnic group were included in the first step. The second step added the interaction between either moderator and TLE. Moderation is confirmed if the second step yields significant increments in *R*^2^, as well as significant differences in estimated conditional means at levels of the moderator [40]. In all regressions, the no trauma group was the referent category. All data were analyzed while using SPSS Version 24, including the PROCESS macro [41] used for mediation (Model 4) and moderation (Model 1) analyses. 

## 3. Results

### 3.1. Descriptive Results

Table 1 presents descriptive statistics. The sample predominantly consists of racial and ethnic minority and low income participants, and it has a slight female majority (59.2%). Blacks, Hispanics, and Asians each constituted at least 20% of the sample. The vast majority of the sample (76.8%) reported household income below $57,782, the median household income of New York City in 2017. The mean age in this college sample was 19.93 (2.46). The majority (78.7%) of participants experienced at least one traumatic life event. This is comparable to lifetime prevalence of 80–90% in previous college samples [42,43] and in adult population-based samples [44,45]. The majority of the sample reported between one and three events (54.5%), with less than a third reporting four or more events (24.1%). 

Figure 1 shows the percentage of the sample that experienced each traumatic life event. The top five events in order were (1) other stressful event, (2) natural disaster, (3) physical assault, (4) sudden death of someone close, and (5) transportation accident. 

There was no significant difference in the proportion of males and females in each TLE group (*χ*^2^(2) = 1.99, *p* = 0.37). There was also no significant difference in TLE by racial and ethnic group *χ*^2^(6) = 11.44, *p* = 0.08. Family income was not significantly different by TLE group either *F*(2813) = 1.59, *p* = 0.21. One-way ANOVA with Bonferroni-corrected post-hoc *t* tests indicated that mean age was significantly different across TLE group *F*(2813) = 4.04, *p* = 0.02. Specifically, participants with 4+ TLE were slightly older (*M* = 20.36, *SD* = 0.20) than the participants with 1–3 TLE (*M* = 19.76, *SD* = 0.11).

Table 1 shows descriptive statistics for PLE by demographics. An independent samples *t*-test found no significant differences in PLE by gender, *t*(814) = 0.08, *p* = 0.94. However, a one-way ANOVA with Bonferroni-corrected post-hoc *t* tests indicated that there were differences by race, with mean PLE scores for Blacks and Asians significantly greater than for Hispanics. A bivariate negative correlation between mean PLE scores and age was significant (*r* = −0.07, *p* = 0.04), while that of the relationship between PLE and income was non-significant (*r* = −0.45, *p* = 0.20).

Bivariate correlations also resulted in significant positive relationships between PLE, dissociation, and ASI, within the entire sample. Mean number of PLE reported was 14.12 (*SD* = 9.17, range: 0–43). The mean dissociation score was 10.35 (*SD* = 10.96, range: 0–76), and the mean ASI score was 11.59 (*SD* = 7.00, range: 0–29). 

### 3.2. Mediation Analysis

**Figure 2:**
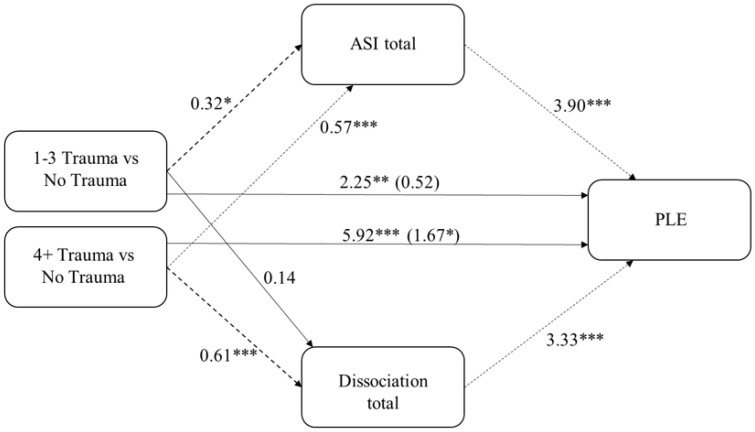
Multicategorical parallel mediation model of the relation between trauma and psychotic-like experiences (PLE) via Aberrant Salience Inventory (ASI) and dissociation. Note: * *p* < 0.05, ** *p* < 0.01, *** *p* < 0.001; unstandardized coefficients reported. Total effects for trauma, not including the mediators in the model appear outside the parenthesis. The Direct effects for trauma, including the mediators in the model, appear inside parenthesis. Mediated paths are presented as dotted lines. Model controls for age, gender, race, and income.

Figure 2 presents the results from the parallel mediation model. The model controls for age, gender, family income, and racial and ethnic group. Asian Americans, women, and the no trauma group are the reference categories. 

The total effect of high cumulative trauma (4+ TLE) on PLE was significant (95% *CI*: 4.08–7.75), as shown by the solid lines in Figure 2, as was the total effect of 1–3 TLE on PLE (95% *CI*: 0.68–3.81). Consistent with a dose response effect, the total effect of 4+ TLE on PLE was larger than the total effect of 1–3 TLE on PLE (*F*(2806) = 20.88, *p* < 0.0001). The total effect of TLE explained 7.4% of the variance in PLE. As indicated by the dotted lines in Figure 2, the indirect effect of both groups of TLE on PLE through ASI was significantly different from zero (1–3 TLE; 95% *CI*: 0.53–2.05; 4+ TLE 95% *CI*: 1.37–3.14). In contrast, the indirect effect via dissociation was only significantly different from zero for 4+ TLE (95% *CI*: 1.27–2.89) and not for 1–3 TLE (95% *CI*: −0.03–1.01). The full model explained 57% of the variance in PLE. The indirect effects via ASI and dissociation were similar in magnitude (difference = −0.074 95% *CI*: (−0.60–0.41)), with both yielding completely standardized indirect effects of 0.08. 

### 3.3. Moderation Analyses

The results for the hierarchical regressions testing the moderating effect of ASI and dissociation appear in Table 2. As shown, the change in *R*^2^ resulting from adding the interaction between ASI and TLE groups was not significant. However, the interactions between the TLE groups and dissociation yielded a significant change in *R*^2^ with only the interaction between 1–3 TLE and dissociation being statistically significant. 

**Figure 3:**
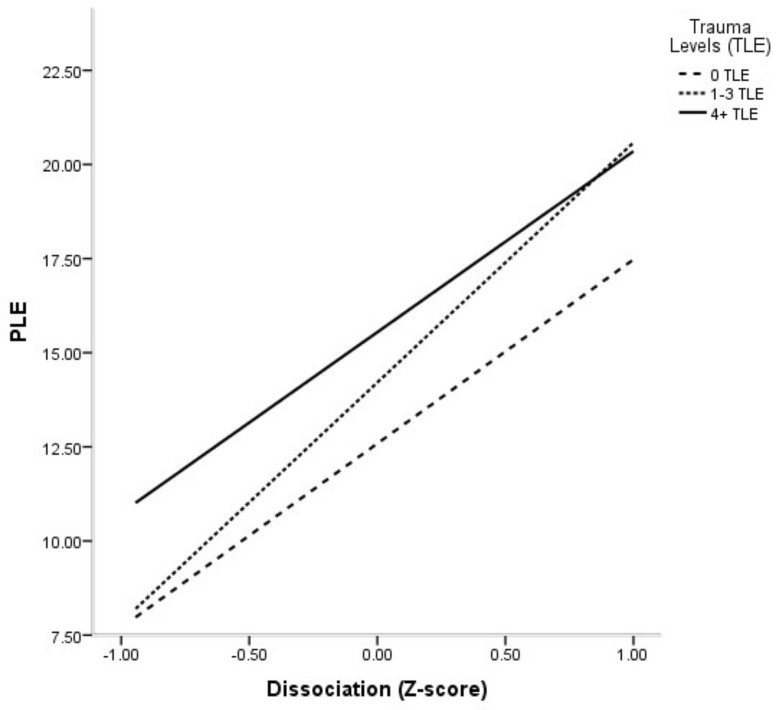
Interaction between dissociation and cumulative trauma levels in predicting PLE.

Figure 3 depicts the estimated relations between dissociation and PLE by cumulative trauma levels. As shown, at low levels of standardized dissociation, PLE was, on average, higher for the high cumulative trauma group (4+ TLE) than for the other two groups (*F*(2803) = 6.20, *p* = 0.002), and PLE, on average, were not statistically different between the no trauma (8.03) and the 1–3 TLE groups (8.27). In contrast, at high standardized levels of dissociation, mean PLE were higher for both the 4+ TLE and the 1–3 TLE groups than for the no trauma group (*F*(2803) = 4.64, *p* = 0.001), with no statistical difference in mean PLE between the high and the 1–3 TLE group. Furthermore, the slopes of all three lines were significantly different from zero for the no trauma (4.77, *p* < 0.001), 1–3 TLE (6.31, *p* < 0.001), and 4+ TLE (4.69, *p* < 0.001) groups, respectively, which indicated that higher dissociation was positively and significantly related to PLE for all trauma groups. Yet, consistent with the significant interaction between dissociation and 1–3 TLE, the expected increase in PLE from higher dissociation was greater in magnitude for the 1–3 TLE group than for all the other groups. 

## 4. Discussion

This study is one of the first to simultaneously investigate the degree to which dissociation and aberrant salience mediated or moderated the relationship between number of different traumatic life events (TLE) and psychotic-like experiences (PLE) in a racially diverse sample. The results showed that the relationship between exposure to TLE and PLE was mediated by aberrant salience, regardless of TLE frequency, and mediated and moderated by dissociation differentially by TLE frequency. Specifically, individuals who experienced a high number of different traumatic life events (four or more) were more likely than those with no trauma to report a higher number of PLE, in part because they experienced more dissociation. For those with a lower frequency of different TLE (i.e., 1–3), the risk for PLE was dependent on the degree of dissociation. For these individuals with 1–3 TLE, if they also reported low levels of dissociation, their mean level of PLE was the same as those who had not experienced any trauma. As dissociation increased, their risk for PLE increased, so much so that high levels of dissociation among this group was associated with mean PLE that was comparable to those who were exposed to a higher number of different traumatic life events. This really suggests that how people respond to or cope with trauma might have a strong impact on their psychosis risk, especially when they have not yet reached the threshold of four or more different TLE. Coping mechanisms may be particularly important after the first exposure of trauma, because it has the potential to disrupt compounding repercussions of the initial trauma [46], and dissociation has been associated with poorer outcomes, even among people in treatment for their PTSD [47]. 

Those exposed to a high number of different traumatic events may have higher mean levels of PLE due to other factors in addition to dissociation, because they have had more time for psychological and neurological changes to occur [48]. Our second main finding was that aberrant salience, a factor that is hypothesized to be important for assessing the emergence of psychosis and psychotic like experiences [20], mediated the relationship between both levels of cumulative traumatic experiences (1–3, 4+) and PLE. The proposed mechanism is that trauma results in a disruption of the Salience Network (SN), a neural system that is implicated in psychosis risk [29]. Perhaps over time, and with repeated exposures to trauma, dysregulation in this salience network is more pronounced.

Evidence from neurological studies indicate that individuals who experience early traumatic stress have a differently organized salience network [49]. The SN is comprised of several brain structures that respond when presented with stimuli that evoke a marked emotional or physical response. In individuals with early exposure to trauma, there is enhanced conectivity in this network, and this increased connectivity could result in changes in information processing, which results in undue attention to otherwise neutral stimuli, or aberrant salience [49]. The idea that repeated exposures could increase dysregulation in the sailence network seems plausible, even though we do not know whether those reporting a higher number of different TLE were exposed earlier and for a longer period of time than those with 1–3 TLE. 

These findings contribute to the literature on aberrant salience by identifying its role as a potential pre-cursor or risk factor for psychotic-like experiences among individuals that were exposed to traumatic events, such as natural disasters, physical assaults, sudden deaths of close loved ones, etc. Previous research has established the mediating role of aberrant salience in the relationship between childhood trauma and psychotic-like experiences [30]. Our research builds on this finding by establishing a broader link between traumatic life events and PLE, and it is consistent with findings from Reininghaus and colleagues [50], who argued that aberrant salience, as well as stress sensitivity and threat anticipation, were important psychological processes in the development of daily psychotic experiences that are present in the early stages of psychotic illness. Importantly, we found that aberrant salience’s role in the relation between trauma and PLE cuts across increasing numbers of different TLE, while controlling for any differences in trauma and PLE due to age, gender, racial and ethnic group, and family income. 

Finally, it is worth revisiting the similarities and differences between aberrant salience and dissociation that emerged as a result of the mediation and moderation analyses. A significant indirect relationship for dissociation was only found for the higher trauma group, while the indirect effects of aberrant salience were significant across the relation between both levels of cumulative trauma and PLE. Moreover, dissociation emerged as a significant moderator of the 1–3 TLE-PLE relationship, while aberrant salience did not. Dissociation, unlike aberrant salience, allows for disconnection from trauma that may also increase the vulnerability to experiencing less connection to other aspects of reality (i.e., PLE). Once a threshold of 4+ different TLE is met, however; dissociation may not further exacerbate psychosis risk, perhaps because some maximum point of association has already been met. In addition, it is possible that other repercussions of experiencing high cumulative trauma enhance psychosis risk, regardless of additional dissociative symptoms. These combined results suggest that while aberrant salience may be more central in explaining the TLE-PLE relationship more broadly, dissociation plays a more dynamic role, operating in parallel with aberrant salience in contexts of high cumulative trauma to account for PLE, while in conditions of lower cumulative trauma, catalyzing the extent to which PLEs may emerge. Revisiting the findings from Reininghaus and colleagues [50], it may be argued that the ambiguity and unpredictability of low trauma contexts in comparison to no or high trauma contexts might result in heightened stress sensitivity, threat anticipation, and aberrant salience, which may result in more PLE if coupled with greater dissociation. A closer look at the interaction of dissociation and the aforementioned phenomena is suggested to better understand this relationship. 

There are several limitations to the present study. First, we used a cross-sectional, correlational design to assess mediation, which limits our conclusions regarding causality and temporal relationships between TLE, PLE, aberrant salience, and dissociation. For example, it is possible that participants’ endorsement of PLE influenced their recall or likelihood of exposure to traumatic life events. However, some of the most frequently endorsed TLE in this sample, e.g., natural disaster, seem less dependent on perception and the measures of PLE that are used in this study assessed current attenuated symptoms. Nevertheless, a prospective longitudinal design similar to Carrion, Weems and Reiss (2007) would increase our ability to conclude that exposure to TLE leads to dysregulation in the salience network that enhances psychosis risk [51]. 

A further limitation of this study may stem from use of the Life Events Checklist (LEC) to measure participants’ exposure to potentially traumatic events. Whereas other studies drew specific links between abuse on one hand and PLEs on the other [30], this study looked for a relationship between psychotic like experiences and a variety of interpersonal and environmental traumatic experiences. The LEC measure that is used to capture cumulative trauma does not explicitly ask about emotional, physical, or sexual abuse, or neglect, which are important predictors of PLE. In addition, the “other stressful” TLE designation was endorsed with the highest frequency in our sample, which suggests that other traumas not readily identifiable in the LEC, possibly including abuse and neglect, may be important contributors to our findings.

Another limitation of this study is the reliance on self-report measures, including for attenuated positive psychotic symptoms, to assess PLE. While the use of this measure captures the portion of the psychotic spectrum that speaks the most to risk in a young adult sample, our findings remain limited by the lack of a clinical interview measure: clinically ill participants, albeit likely few, may have remained undetected, while individuals not at risk for psychosis may have been detected by the present study. Notwithstanding, psychotic symptoms occur on a continuum [52] and subclinical attenuated symptoms play a role in the etiology of psychotic syndromes [52,53]; and, they are often part of the disorder profile of other common mental disorders. Additionally, the concerns about self-report methods are somewhat tempered by the strong psychometric properties of the validated measures that were used in the present study. 

While our sample comprises college students, which limits generalizability, it is less vulnerable to the selection biases characteristic of “traditional” college samples. While our findings may not generalize to Whites with no immigration history, this sample was selected from a public university that primarily serves racial and ethnic minority working class individuals in the surrounding city—an ideal population to study the associations to traumatic life events. Our observed mean differences in PLE across frequency of trauma were over and above any differences in TLE due to age, gender, family income, or specific racial and ethnic group. Nevertheless, future research should examine whether these findings can be generalized to nationally representative U.S. samples and to White majority samples.

In conclusion, our results provide evidence that dissociation and aberrant salience are both important factors that help to explain the association between the frequency of traumatic life events (TLE) and psychotic like experiences (PLE) in a racially and ethnically diverse sample of young non-treatment seeking adults. Moreover, dissociative symptoms seemed to catalyze the association between trauma and PLE for people who have not quite reached the 4+ TLE threshold. This suggests that more active ways of coping with initial trauma should be encouraged among emerging adults and, especially, college students of color who according to our results, have a high likelihood of being exposed to at least one TLE. While the relationship between exposure to childhood trauma, dissociation, and delusions and hallucinations has been explored in prior research [12,13,14,15], this research establishes a broader link by exploring dissociation and PLE, a part of the broader psychotic spectrum. Early intervention efforts that are designed to alleviate the psychological impact of trauma among young people should use interview tools that inquire about the history of various types and frequency of trauma exposure (environmental, etc.). The timely processing of such trauma (providing ample resources for those who need it, crisis phone lines, counseling services, etc.) seems like an important implication, especially within the context of natural disasters or other environmental TLE that a non-treatment-seeking sample would be more likely to experience. Interventions that alleviate the psychological impact of trauma, and that subsequently reduce the risk for dissociation or aberrant salience, may reduce the risk of PLE for individuals who need intervention. Lastly, this study highlights the need for more research and advocacy of marginalized individuals in high trauma environments, as it may provide better insight into how individuals who experience multiple traumas cope with these types of events. These efforts could reduce the risk for PLE in such individuals. 

## Figures and Tables

**Table 1:** Sample Demographics and Characteristics.

	Frequency *n* = 816	Percent
**Race**		
Black	176	21.6%
Asian	257	31.5%
Hispanic	248	30.4%
Other	135	16.5%
**Gender**		
Female	483	59.2%
Male	333	40.8%
**Trauma**		
0 TLEs	174	21.3%
1–3 TLEs	445	54.6%
≥4 TLEs	197	24.1%
**Income Level**		
≤17,000	220	27.0%
17,000–25,000	190	23.3%
25,000–50,000	216	26.4%
≥50,000	190	23.3%
	**Mean (SD)**	
**PLE by Gender**		
Female	14.14 (9.06)	
Male	14.09 (9.33)	
**PLE by Race**		
Black	15.35 (8.67)	
Asian	15.25 (9.64)	
Hispanic	12.55 (8.56)	
Other	13.24 (9.52)	
**PLE by Income Level**		
≤17,000	14.21 (9.07)	
17,000–25,000	15.17 (9.49)	
25,000–50,000	14.41 (9.39)	
≥50,000	12.63 (8.56)	

**Table 2:** Multicategorical hierarchical regression models for the interactions between dissociation and TLE and ASI and TLE predicting PLE.

	Dissociation Model			ASI Model		
	*b*	*SE*	*B*	*b*	*SE*	*B*
Age	−0.09	0.10	−0.03	−0.06	0.10	−0.02
Male	0.10	0.55	0.01	−0.17	0.48	−0.01
Family Income	−0.09	0.06	−0.04	−0.09	0.06	−0.04
Black	0.33	0.71	0.02	−0.34	0.67	−0.02
Hispanic	−**1.53**	**0.64**	−**0.08**	−**1.19**	**0.60**	**0.06**
Other	−1.36	0.77	−0.06	−0.18	0.72	−0.01
TLE 1–3	**1.69**	**0.66**	**0.09**	0.61	0.62	0.03
TLE 4+	**3.04**	**0.78**	**0.14**	**2.80**	**0.73**	**0.13**
Dissociation	**4.77**	**0.64**	**0.52**	-	-	-
Dissociation * 1–3 TLE	**1.54**	**0.75**	**0.11**	-	-	-
Dissociation * 4+ TLE	−0.08	0.75	−0.01	-	-	-
ASI				**5.59**	**0.55**	**0.61**
ASI * 1–3 TLE				0.15	0.63	0.01
ASI * 4+ TLE				1.20	0.73	0.07
Constant	**16.29**			**16.03**		
Adjusted *R*^2^	**0.41**			**0.47**		
*F*-value Change in *R*^2^	**4.74**			1.97		

Note: Asian is the reference group for race/ethnicity; **bold** = ***p*** < **0.05**. Model controls for age, gender, race and ethnicity and family income. * indicates multiplicative term.
